# Ferryl Hemoglobin and Heme Induce A_1_-Microglobulin in Hemorrhaged Atherosclerotic Lesions with Inhibitory Function against Hemoglobin and Lipid Oxidation

**DOI:** 10.3390/ijms22136668

**Published:** 2021-06-22

**Authors:** Dávid Pethő, Tamás Gáll, Zoltán Hendrik, Annamária Nagy, Lívia Beke, Attila Péter Gergely, Gábor Méhes, Csaba Tóth, Magnus Gram, Bo Åkerström, György Balla, József Balla

**Affiliations:** 1 Division of Nephrology, Department of Internal Medicine, Faculty of Medicine, University of Debrecen, 4032 Debrecen, Hungary; petho.david@med.unideb.hu (D.P.); gall.tamas@med.unideb.hu (T.G.); nagy.annamari90@gmail.com (A.N.); 2 Kálmán Laki Doctoral School, Faculty of Medicine, University of Debrecen, 4032 Debrecen, Hungary; 3 ELKH-UD Vascular Biology and Myocardial Pathophysiology Research Group, Hungarian Academy of Sciences, University of Debrecen, 4032 Debrecen, Hungary; balla@med.unideb.hu; 4 Department of Forensic Medicine, Faculty of Medicine, University of Debrecen, 4032 Debrecen, Hungary; dr.hendrik.zoltan@gmail.com (Z.H.); gergely.peter@med.unideb.hu (A.P.G.); 5 Department of Pathology, Faculty of Medicine, University of Debrecen, 4032 Debrecen, Hungary; beke.livia@med.unideb.hu (L.B.); gabor.mehes@med.unideb.hu (G.M.); 6 Division of Vascular Surgery, Department of Surgery, Faculty of Medicine, University of Debrecen, 4032 Debrecen, Hungary; toth.csaba@med.unideb.hu; 7 Department of Clinical Sciences Lund, Pediatrics, Lund University, 22184 Lund, Sweden; magnus.gram@med.lu.se; 8 Department of Clinical Sciences Lund, Infection Medicine, Lund University, 22184 Lund, Sweden; bo.akerstrom@med.lu.se; 9 Department of Pediatrics, Faculty of Medicine, University of Debrecen, 4032 Debrecen, Hungary

**Keywords:** atherosclerosis, hemorrhage, α_1_-microglobulin, hemoglobin oxidation, heme, Ferryl Hemoglobin, oxidized lipoprotein

## Abstract

Infiltration of red blood cells into atheromatous plaques and oxidation of hemoglobin (Hb) and lipoproteins are implicated in the pathogenesis of atherosclerosis. α_1_-microglobulin (A1M) is a radical-scavenging and heme-binding protein. In this work, we examined the origin and role of A1M in human atherosclerotic lesions. Using immunohistochemistry, we observed a significant A1M immunoreactivity in atheromas and hemorrhaged plaques of carotid arteries in smooth muscle cells (SMCs) and macrophages. The most prominent expression was detected in macrophages of organized hemorrhage. To reveal a possible inducer of A1M expression in ruptured lesions, we exposed aortic endothelial cells (ECs), SMCs and macrophages to heme, Oxy- and FerrylHb. Both heme and FerrylHb, but not OxyHb, upregulated A1M mRNA expression in all cell types. Importantly, only FerrylHb induced A1M protein secretion in aortic ECs, SMCs and macrophages. To assess the possible function of A1M in ruptured lesions, we analyzed Hb oxidation and heme-catalyzed lipid peroxidation in the presence of A1M. We showed that recombinant A1M markedly inhibited Hb oxidation and heme-driven oxidative modification of low-density lipoproteins as well plaque lipids derived from atheromas. These results demonstrate the presence of A1M in atherosclerotic plaques and suggest its induction by heme and FerrylHb in the resident cells.

## 1. Introduction

Hemeproteins play vital roles in oxygen and electron transports, as well as in oxidation–reduction enzyme reactions. Hemoglobin (Hb), responsible for oxygen transport, is the most abundant hemeprotein in humans. Hb oxidation is a common phenomenon not only in pathological states but also in physiological conditions [1,2,3,4]. Importantly, Hb undergoing a natural auto-oxidation in red blood cells (RBCs) generates reactive oxygen species (ROS) and hydrogen peroxide (H_2_O_2_) [5,6,7]. In intact RBCs, Hb is protected from oxidative modification by a complex antioxidant system including metHb reductase, glutathione, superoxide dismutase, catalase, glutathione peroxidase and glutathione reductase [8,9]. Surprisingly, the rate of Hb auto-oxidation increases in the microcirculation as the consequence of lower partial oxygen pressure compared to the higher arterial oxygen concentration [7]. In vascular pathological conditions, such as atherosclerosis with intraplaque hemorrhage [10], subarachnoid hemorrhage [11], sickle cell disease [12] and sepsis [13], Hb is liberated from RBCs and accumulates as cell-free Hb in tissues out of control from the cellular antioxidant defense system.

An atherosclerotic plaque is classified as a complicated lesion when the atheroma is infiltrated by RBCs [14] due to plaque rupture or the leakage of the immature, intra-plaque neovascularized capillaries [15,16,17]. In the oxidizing milieu of atherosclerotic plaques, RBCs easily lyse followed by Hb release [10]. Cell-free Hb then triggers a variety of adverse effects resulting in cell and tissue damage [18].

Reactions of Hb with ROS have been thoroughly studied. The interaction between Hb and free radicals generates oxidized Hb forms characterized mainly by the oxidation state of the iron in the heme group [19]. The oxidation state of iron in OxyHb is ferrous (Fe^2+^); one-electron oxidation of the heme group in Hb results in MetHb (Fe^3+^) and the loss of two electrons leads to FerrylHb (Fe^4+^ = O^2−^) formation. FerrylHb can be reduced back to MetHb by reductants or self-reduction performed by globin [20]. The ferrous form of the heme group has been described to react with nitric-oxide, reducing its vasodilator effect and leading to vasoconstriction, hypertension and MetHb formation [21]. Hb also facilitates hydroxyl-radical generation from activated oxygen species via the Fenton reaction [22]. Furthermore, Hb reacts with plaque lipids in the atheroma, leading to Hb oxidation and formation of MetHb and FerrylHb species [10]. Globin radicals found in Hb create covalently cross-linked Hb dimers and multimers which are present in human complicated atherosclerotic lesions [10]. A devastating consequence of Hb oxidation is the release of free heme from the globin [23] that further aggravates Hb toxicity [24]. Besides, FerrylHb exerts a pro-inflammatory effect activating nuclear factor kappa-light-chain-enhancer of activated B cells (NFΚB) [25] and NLR family pyrin domain-containing 3 (NLRP3) inflammasome [26]. Recently, it was reported that Hb oxidation generates globin-derived peptides, triggering endothelial damage and inducing tumor necrosis factor-α and interleukin-1β expression [26]. Importantly, both cell-free Hb and heme released from oxidized Hb catalyze low-density lipoprotein (LDL) oxidation [27,28,29,30].

Natural defense proteins against Hb- and heme-induced toxicity are haptoglobin (Hp), hemopexin (Hpx), the antioxidant and heme-scavenger protein α_1_-microglobulin (A1M) and the intracellular heme-catabolizing heme oxygenase-1 (HO-1)/ferritin system. Hp stabilizes Hb structure and protects against the oxidative reactions of Hb with lipoproteins [31]. Hb–Hp complexes are then taken up by macrophages via their CD163 receptors, followed by lysosomal degradation of Hb–Hp complexes and the degradation of heme by the intracellular heme catabolizing enzyme HO-1 [32,33]. Free plasma heme is specifically scavenged by the acute-phase protein Hpx [34], or, when it is taken up by cells, heme is degraded by HO-1 [35].

A1M is a small (26 kDa) protein belonging to the lipocalin family [36]. A1M is predominantly synthesized in the liver and associates with a variety of proteins including IgA, albumin, or prothrombin [37]. However, due to a rapid distribution via blood and a low grade biosynthesis in all nucleated cells, it can be detected in many cells and tissues, including skin, intestines, placenta and kidneys [38,39]. A1M has been shown to be an efficient radical scavenger and heme-binding protein [40,41].

Since Hb oxidation derivatives and free heme play an essential role in atherosclerotic plaque development, we suggest that heme-binding and antioxidant protein A1M might be involved in the protection of the arterial wall against Hb/heme-driven damage. In this study, we investigated the presence of A1M in different stages of atherosclerotic plaques, such as non-complicated atheromas and complicated plaques with acute bleeding, as well as organized hemorrhage. In cell culture models, we examined the A1M-producing capability of the resident cells of the vessel wall by exposing human aorta endothelial cells (HAoECs), human aortic smooth muscle cells (HAoSMCs) and peripheral blood mononuclear cells (PBMCs) to heme, OxyHb and FerrylHb. Finally, we investigated the protective properties of recombinant A1M (rA1M) in Hb oxidation and heme-driven lipid peroxidation that are hallmarks of atherosclerosis progression.

## 2. Results

### 2.1. A1M Protein Is Abundantly Present in Human Carotid Artery Plaques

**Figure 1:**
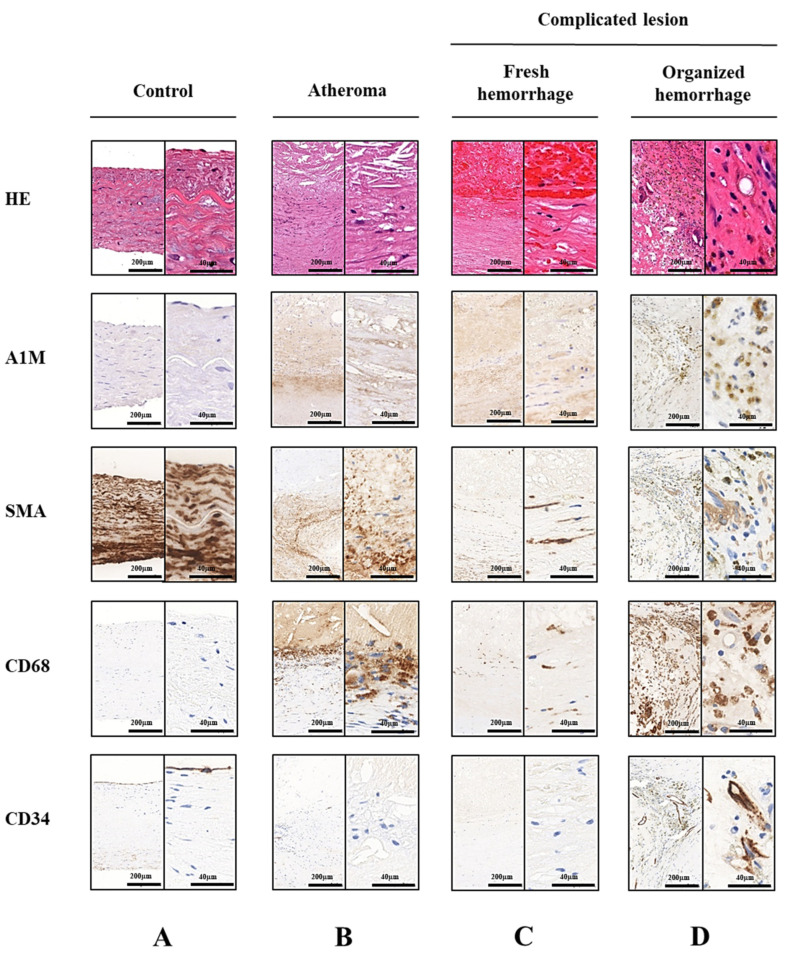
Localization of α_1_-microglobulin (A1M) in healthy carotid wall, atheromatous plaque and complicated lesion with fresh or organized hemorrhage. Serial sections of healthy carotid wall (Panel (**A**)), atheromatous plaque (Panel (**B**)), complicated lesion with fresh (Panel (**C**)), or organized hemorrhage (Panel (**D**)) are shown. Smooth muscle cells were identified by smooth muscle α-actin (SMA), macrophages by CD68 and endothelial cells by CD34 stainings. Red blood cells were visualized by hematoxylin-eosin (HE) staining.

A1M is a general tissue cleaning protein with specific functions—scavenging heme and free radicals [42]. Intraplaque hemorrhage, which is a life-threatening event during atherosclerotic plaque progression, results in Hb release from extravasated RBCs with subsequent Hb oxidation that triggers cell and tissue damage, further destabilizing the plaque [15,17,43]. To examine whether hemorrhage affects the presence of A1M in atherosclerotic plaques, we analyzed healthy carotid arteries, atheromas and carotid artery plaques with acute and organized hemorrhage by immunohistochemistry in human carotid endarterectomy specimens. To analyze the cellular distribution of A1M, smooth muscle cells were identified by smooth muscle α-actin (SMA), macrophages by CD68, while endothelial cells by CD34 staining. Healthy carotid arteries (Figure 1A) showed the characteristics of physiologic smooth muscle cells (SMCs) and extracellular matrix arrangement, a low number of inflammatory cells and no signs of lipid accumulation. Healthy carotid arteries showed weak A1M-specific staining that was mostly observed in the subendothelial region. Non-complicated plaques (Figure 1B) were characterized by extensive extracellular lipid accumulation and foam cell formation, together with distorted SMC arrangement. A1M labeling was increased not only in the vascular extracellular space but both in SMCs and macrophages as well. In the case of acute plaque hemorrhages (Figure 1C), the presence of intact RBCs was detected by hematoxylin-eosin staining. In these lesions, a more pronounced A1M positivity could be observed compared to the non-complicated atheromas. Moreover, A1M was accumulated mostly in macrophages closest to the acute hemorrhage, as well as in the surrounding SMCs. In organized hemorrhage (Figure 1D), intact RBCs could not be detected by hematoxylin-eosin staining. Compared to the other lesions, we found the most significant A1M reactivity in the macrophages of the lesions with organized hemorrhage.

### 2.2. Heme and FerrylHb Induce A1M Expression in Human Aortic Endothelial Cell-, Human Aortic Smooth Muscle Cell- and Peripheral Blood Mononuclear Cell Cultures

**Figure 2:**
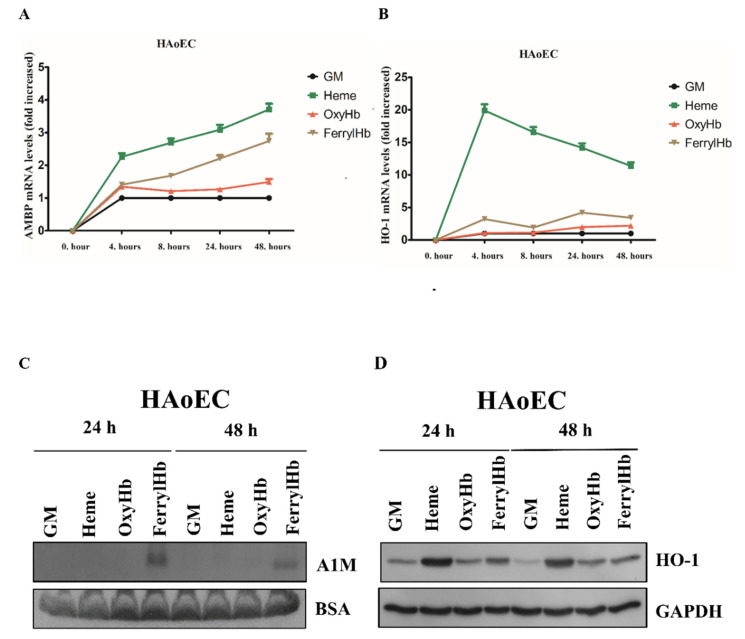
A1M expression is induced in human aortic endothelial cells (HAoECs). HAoECs were exposed to heme (10 µM), OxyHb (10 µM), or FerrylHb (10 µM) in the presence of 5% foetal calf serum. A1M (**A**) and HO-1 (**B**) mRNA expression was quantified by qPCR after 4, 8, 24 and 48 h. (**C**) Secreted A1M protein was detected by immunoblotting in cell culture supernatants, while HO-1 protein level (**D**) was examined in cell lysates after 24 and 48 h. GM: growth medium, BSA: bovine serum albumin, GAPDH: glyceraldehyde 3-phosphate dehydrogenase.

Since A1M is predominantly produced in the parenchymal cells of the liver and secreted into the blood, we next asked whether A1M detected in human atherosclerotic plaques originates from the liver/circulation, or, at least in part, whether it can be synthesized by the resident cells of the vessel wall. As described previously, Hb and its oxidation products heme and FerrylHb are abundantly present in hemorrhaged atherosclerotic plaques [10,18]. Given that intensive A1M positivity was detected in hemorrhaged carotid artery specimens, we examined whether OxyHb, FerrylHb and heme induce A1M expression in the resident cells of atherosclerotic plaques; thus, HAoECs, HAoSMCs and PBMCs were exposed to heme (10 µM), OxyHb (10 µM), or FerrylHb (10 µM). A1M mRNA expression was quantified by quantitative reverse-transcription PCR, while A1M protein secretion was detected by immunoblotting in the cell culture supernatants. We showed that both heme and FerrylHb but not OxyHb induced A1M mRNA expression in HAoECs (Figure 2A).

Expression of HO-1 mRNA, the primary heme-catabolizing enzyme, was also significantly induced by heme and slightly by FerrylHb (Figure 2B). Secreted A1M protein was detected only in FerrylHb-treated groups (Figure 2C). HO-1 protein was induced by heme, FerrylHb and OxyHb, still present in cells at both 24 and 48 h (Figure 2D).

**Figure 3:**
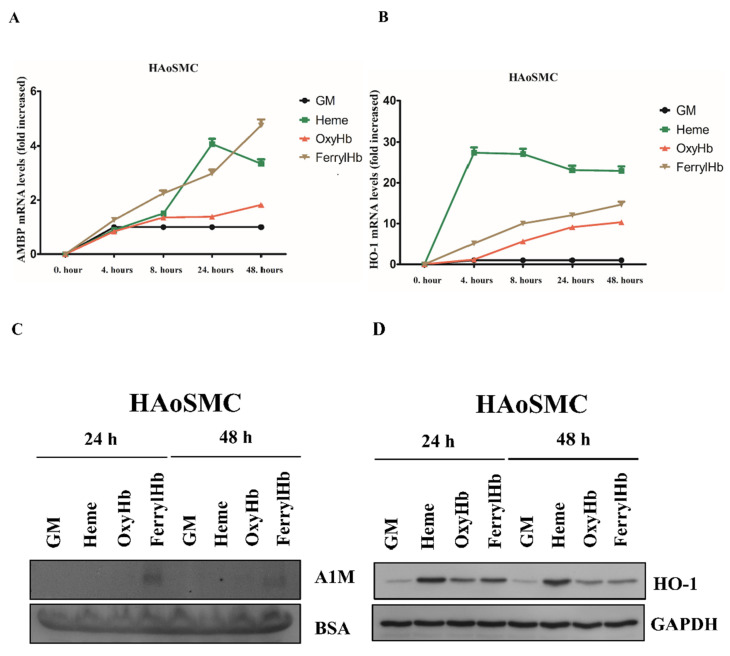
A1M expression is induced in human aortic smooth muscle cells (HAoSMCs). HAoSMCs were exposed to heme (10 µM), OxyHb (10 µM), or FerrylHb (10 µM) in the presence of 5% foetal calf serum. A1M (**A**) and HO-1 (**B**) mRNA expression was quantified by qPCR after 4, 8, 24 and 48 h. (**C**) Secreted A1M protein was detected by immunoblotting in cell culture supernatants, while HO-1 protein expression (**D**) was examined in cell lysates after 24 and 48 h. GM: growth medium, BSA: bovine serum albumin, GAPDH: glyceraldehyde 3-phosphate dehydrogenase.

Since A1M positivity was detected in SMCs surrounding the hemorrhage, we examined whether HAoSMCs express A1M in vitro. Similar to endothelial cells, A1M mRNA was significantly induced by both heme and FerrylHb and slightly by OxyHb in a time-dependent manner (Figure 3A).

HO-1 mRNA was also induced by heme, OxyHb and FerrylHb (Figure 3B). Secreted A1M protein was only detected when cells were exposed to FerrylHb (Figure 3C). Similar to endothelial cells, HO-1 protein was also detectable in cells exposed to heme, OxyHb and FerrylHb at both 24 and 48 h (Figure 3D).

**Figure 4:**
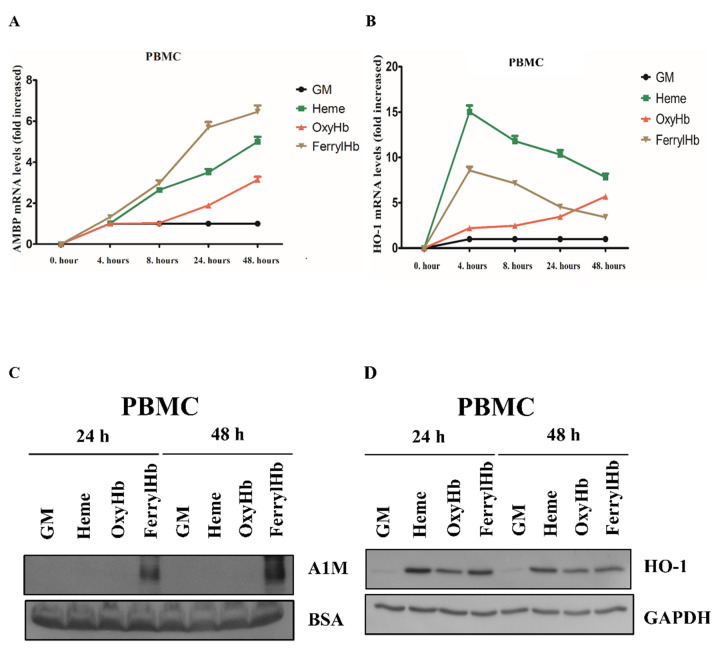
A1M expression is induced in human peripheral blood mononuclear cells (PBMCs). PBMCs were exposed to heme (10 µM), OxyHb (10 µM), or FerrylHb (10 µM) in the presence of 5% foetal calf serum. A1M (**A**) and HO-1 (**B**) mRNA expression was quantified by qPCR after 4, 8, 24 and 48 h. (**C**) Secreted A1M protein was detected by immunoblotting in cell culture supernatants, while HO-1 protein expression (**D**) was examined in cell lysates after 24 and 48 h. GM: growth medium, BSA: bovine serum albumin, GAPDH: glyceraldehyde 3-phosphate dehydrogenase.

As macrophages showed strong A1M positivity in hemorrhaged atherosclerotic plaques, we examined whether PBMCs express A1M in vitro. We showed that A1M mRNA was strongly induced by heme and FerrylHb and slightly by OxyHb in a time-dependent manner in PBMCs (Figure 4A).

HO-1 mRNA was also significantly induced by heme and FerrylHb, whereas OxyHb displayed a somewhat different time course in the increase in HO-1 expression (Figure 4B). Secreted A1M protein was only detectable in cells exposed to FerrylHb (Figure 4C). HO-1 protein was markedly induced in cells exposed to heme, OxyHb and FerrylHb at both 24 and 48 h (Figure 4D). Overall, we can conclude that resident vascular wall cells can synthesize A1M, which raises the possibility that A1M can be, at least in part, locally produced in atherosclerotic lesions in response to FerrylHb and heme.

### 2.3. rA1M Inhibits Hb Oxidation

**Figure 5:**
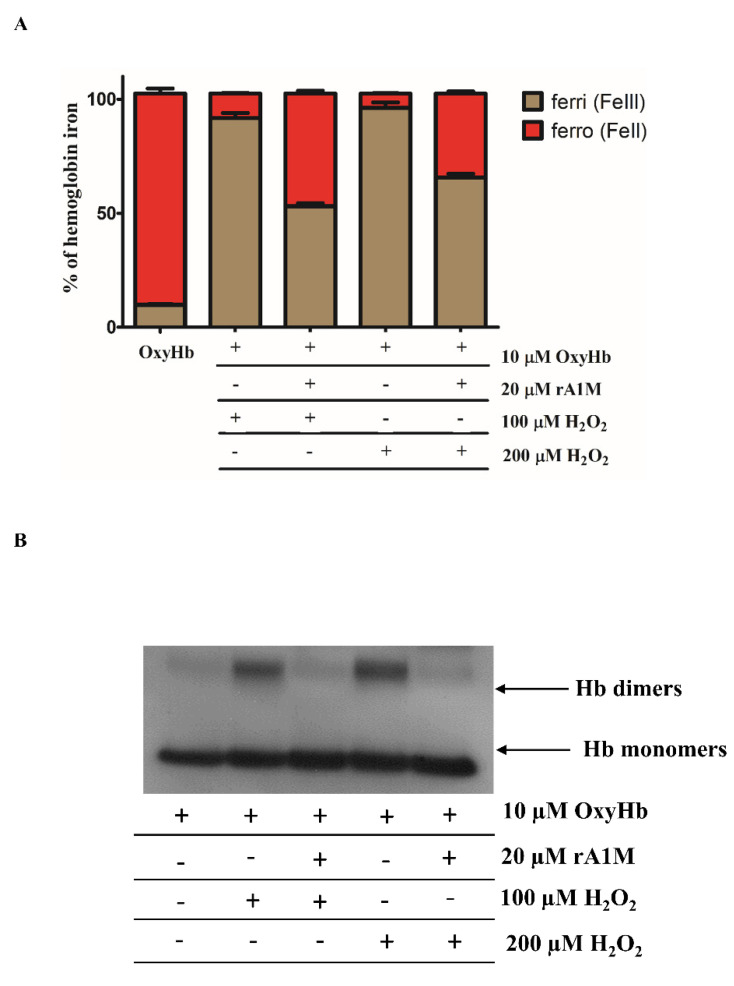
Recombinant A1M (rA1M) inhibits hemoglobin oxidation. Purified OxyHb (10 µM) was incubated for 3 h at 37 °C with 100 or 200 µM of H_2_O_2_ in the presence or absence of rA1M (20 µM). (**A**) Amounts of different redox states of Hb were determined by analysis of the visible spectra. The presence of different redox states of Hb as a percentage of total heme was calculated. (**B**) Representative immunoblot showing covalently cross-linked Hb multimers after Hb oxidation.

Hb oxidation is implicated in the pathogenesis of atherosclerotic plaque progression [18], as well as in other hemolytic diseases [11,12,13,44]. Having shown that A1M is induced by the Hb oxidation products heme and FerrylHb, we next examined whether A1M inhibits the process of Hb oxidation. Hb (10 µM) was exposed to 100 µM and 200 µM concentrations of H_2_O_2_ for 3 h at 37 °C in the presence or absence of rA1M (20 µM). The oxidation process of Hb was followed by measuring the oxidation states of the heme-iron. The quantity of the oxidation forms of iron was determined spectrophotometrically, as summarized in the Methods section. Absorption spectra are added as Supplementary Materials. OxyHb represents ferrousHb which can carry oxygen. H_2_O_2_ (100 and 200 µM) catalyzed the rapid oxidation of Hb forming MetHb; the proportion of this oxidized form reached 90% of total Hb (Figure 5A). MetHb formation was markedly inhibited by rA1M displaying a decrease in metHb formation with about 30–40% depending on the H_2_O_2_ concentration (Figure 5A). Next, we used immunoblotting to analyze cross-linked Hb as a marker of Hb oxidation. Exposure to H_2_O_2_ treatment alone resulted in significant oxidation of Hb reflected by Hb dimer formation that was significantly inhibited by A1M (Figure 5B).

### 2.4. rA1M Inhibits Heme-Induced of LDL/Plaque Lipid Peroxidation and Subsequent Cell Death

**Figure 6:**
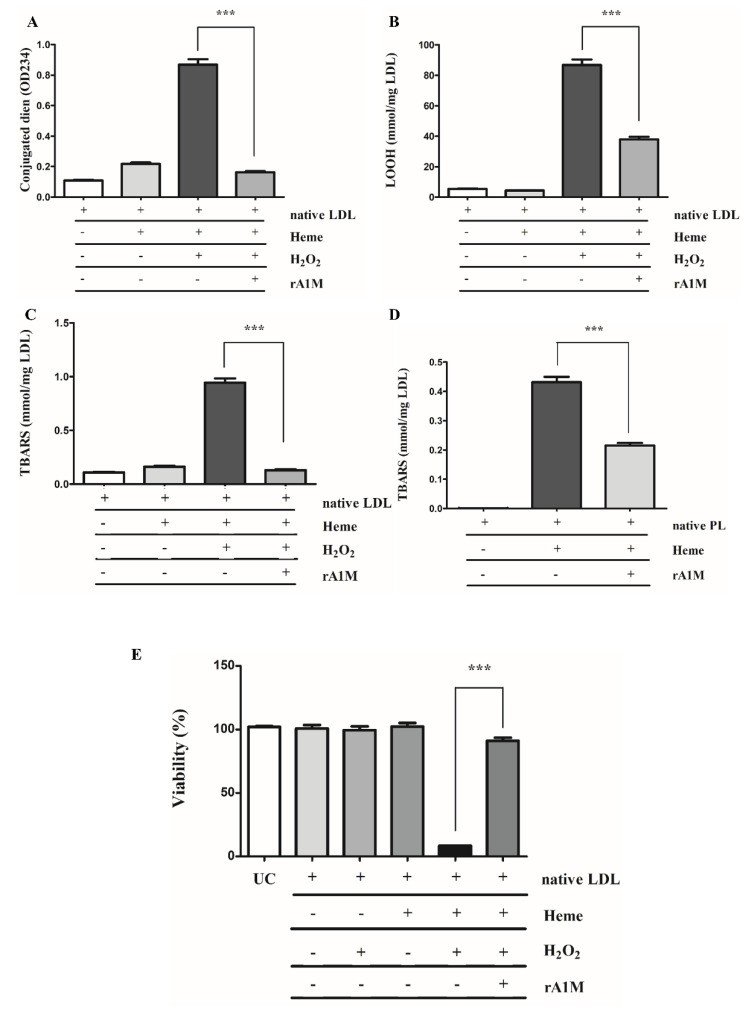
Recombinant A1M (rA1M) inhibits heme-catalyzed oxidation of low-density lipoproteins (LDLs) and plaque lipids as well as oxidized LDL-induced cell death. Native LDLs (200 µg/mL) were incubated alone or in the presence of heme (5 µM), heme (5 µM) and H_2_O_2_ (75 µM), or heme (5 µM) and H_2_O_2_ (75 µM) and A1M (10 µM) in Hank’s balanced salt solution. Conjugated diene (**A**), total lipid hydroperoxides (LOOH) (**B**) and thiobarbituric-acid reactive substances (TBARs) (**C**) were measured. (**D**) Plaque lipids (500 µg/mL), were incubated with heme (5 µM) in the presence or absence of A1M (10 µM) for 3 days at 37 °C. Lipid peroxidation was assessed by measuring TBARs. (**E**) Confluent HAoECs grown in 96-well tissue-culture plates were washed twice with HBSS+ and exposed to native LDLs (200 µg/mL), native LDLs (200 µg/mL) and heme (5 µM), native LDLs (200 µg/mL) and H_2_O_2_ (75 µM), or native LDLs (200 µg/mL), heme (5 µM) and H_2_O_2_ (75 µM) in the presence of A1M (10 µM) in HBSS+. Cells were then incubated at 37° for 6 h in a CO_2_ incubator. Cell viability was assessed by MTT assay. UC: untreated cells. *** *p* < 0.001.

We have previously shown that heme mediates LDL oxidation leading to endothelial cell death [27]. To model the interaction between heme and LDLs in hemorrhaged atherosclerotic plaques, native LDLs (nLDLs; 200 µg/mL) were incubated with heme (5 µM) and H_2_O_2_ (75 µM) in the presence or absence of A1M (10 µM). Oxidative modification of LDLs was analyzed by measuring the concentrations of conjugated dienes (Figure 6A), lipid hydroperoxides (LOOHs) (Figure 6B) and thiobarbituric-acid reactive substances (TBARs) (Figure 6C). We showed that incubation of nLDLs with heme and H_2_O_2_ dramatically increased the formation of lipid peroxidation products that was significantly inhibited by A1M (Figure 6A–C).

Next, we examined whether A1M decreases the peroxidation of lipids isolated directly from human atherosclerotic plaques. Native plaque lipids (500 µg/mL) were incubated with heme (5 µM) in the presence or absence of A1M (10 µM) followed by TBARs measurement. This showed that heme triggered robust plaque lipid peroxidation that was significantly attenuated by A1M (Figure 6D).

Given that heme-oxidized LDLs are highly toxic to endothelial cells, next we asked whether A1M attenuates cytotoxicity mediated by heme-oxidized LDLs. We showed that LDL oxidation by heme and H_2_O_2_ dramatically reduced cell viability compared to either LDLs and heme or LDLs and H_2_O_2_. Importantly, the presence of A1M in the highly oxidizing milieu (LDLs, heme and H_2_O_2_) preserved endothelial cell viability (Figure 6E) supporting the possible protective role of A1M.

## 3. Discussion

Oxidation of Hb [10,26] and lipoproteins in the artery wall [45,46] play a vital role in the progression of atherosclerosis. Intraplaque hemorrhage is a life-threatening event during atherosclerotic plaque progression [15,17,43] resulting in Hb release from extravasated RBCs. Hb oxidation triggers cell and tissue damage, further destabilizing the plaque. Therefore, potential strategies targeting the oxidation of Hb and plaque lipids generate therapeutic interest. A1M has been shown to have significant reductase, radical-scavenging and heme-binding properties (reviewed by Kristiansson et al.) [47]. Based on this complex function, we postulate that A1M is present in atherosclerotic plaques, particularly in their hemorrhaged complicated states, and A1M may be induced locally and represents a protective response in the resident cells of the artery wall.

To examine A1M localization in different stages of atherosclerosis, first, we analyzed the presence of A1M in healthy carotid arteries, as well as in atheromas and hemorrhaged plaques derived from patients who had undergone carotid endarterectomy. Our first aim was to find a possible correlation between plaque progression and the presence of A1M. According to this, healthy carotid arteries showed a weak sub-endothelial A1M localization by immunohistochemistry, while atheromas and hemorrhaged plaques were characterized by robust A1M positivity. The most intensive A1M staining was observed in atherosclerotic plaques with organized hemorrhage. Given that both oxidative stress [48,49] and hemorrhage [10,18] play a significant role in atherosclerosis, it is reasonable to assume that A1M, due to its pronounced radical-scavenging and heme-binding, represents a protective response to plaque progression. The presence of A1M in the arterial wall was recently also demonstrated with mouse aortas using immunofluorescence [50]. In this report, a strong A1M-staining was seen on the luminal surface of the endothelium, besides a sub-endothelial localization, and the A1M-activity was co-localized with heparan-sulfate.

Since A1M is predominantly synthesized in the liver [51] and secreted to the blood-stream, followed by rapid distribution to extravascular compartments, it is reasonable to assume that the main source of A1M in the vessel wall is the hepatic parenchymal cells [39,52]. However, since A1M mRNA has been detected in most other human cell types besides the liver (reviewed in [47]), next we examined whether the resident cells of atherosclerotic plaques close to the organized hemorrhage can synthesize A1M in response to Hb and its oxidation products. In order to exclude a possible liver origin, we evaluated this as cell culture models. Therefore, we examined whether Hb or its oxidation products heme and FerrylHb induce A1M expression in the resident cells of atherosclerosis in vitro—those are human aortic endothelial cells (HAoECs), human aortic smooth muscle cells (HAoSMCs) and macrophages. Here we showed that heme induces A1M mRNA expression in cell cultures, supporting previous results in human primary renal proximal tubule epithelial cells [53] and keratinocytes [54]. Whereas A1M protein levels remained below the detection limit when cells were exposed to free heme, FerrylHb induced a significant A1M secretion in all cell types. Overall, our results demonstrated that A1M detected in human carotid plaques might originate, at least partly, from the resident cells of atherosclerosis.

Previous studies have shown that both Oxy- and MetHb induce A1M protein secre-tion in HepG2 hepatoma cells [55]; A1M secretion is also induced by MetHb in U937 histiocytic and K562 erythroid cell lines [55]. Unlike HepG2 hepatoma cells [55], we did not observe that OxyHb induced a considerable A1M mRNA expression in HAoECs and HAoSMCs. However, it did induce an A1M mRNA expression in PBMCs after 24 and 48 h. This might be explained by the data of Schaer, demonstrating that Hb is taken up via the macrophage scavenger receptor CD163 in the absence of Hp [56]. Importantly, we found that FerrylHb induced an A1M mRNA expression more effectively, compared to OxyHb. In the present study, we also followed A1M secretion by immunoblot, which showed that only FerrylHb, but not OxyHb, induced A1M protein secretion to the experimental medium. Another important finding of our study was that FerrylHb, but not OxyHb, induced A1M secretion in HAoECs, HAoSMCs and PBMCs, suggesting a possible unique nature of FerrylHb compared to OxyHb. This could, at least in part, be explained by the transient nature of FerrylHb, which enables it to release its heme moieties [57], acts as a source of free radicals and exhibits a modified globin chain. Although Hb can also be a source of free radicals, the rate of free radical generation from FerrylHb is more prominent. This is, in fact, supported by our recent study demonstrating that FerrylHb, but not OxyHb, inhibits osteoclastic differentiation of macrophages both in vitro and in hemorrhaged atherosclerotic plaques [58], suggesting a unique characteristic of FerrylHb in atherosclerotic plaque development. The contrast between hepatoma cell and vessel wall cells could be explained by the different experimental setups. HepG2 cells, representing a major source of A1M production, secrete a considerable amount of A1M without Hb stimulus [55] compared to our cell cultures. In addition, HepG2 cells were exposed to Hb in serum-free conditions for 1–6 h, while, in our experiments, cells were incubated in the presence of serum (5%) for 24 and 48 h [55]. Another possible explanation could be that oxidation-induced A1M expression is regulated by nuclear factor erythroid 2-related factor 2 (Nrf2) [59,60], the master regulator of antioxidant gene expression, among them, HO-1 [61]. As HO-1 plays an indispensable role to mitigate both Hb- [62] and heme-induced toxicity [63], we postulate that HO-1 activation decreases heme- as well as OxyHb-induced stress levels lowering A1M protein secretion below the detection limit.

The gene encoding the A1M protein is designated as α1-microglobulin-bikunin precursor gene (AMBP), encoding the A1M and bikunin proteins [64]. In the liver, transcription of the AMBP gene is regulated by hepatocyte nuclear factors (HNFs) [65]. AMBP expression is also regulated by the Keap-1/Nrf-2 pathway in multiple cell lines [59,66]; however, data on the regulation of AMBP gene expression in primary cells are lacking. The contrast between detectable AMBP mRNA and undetectable A1M protein expression in ECs, SMCs and PBMCs exposed to heme and OxyHb might indicate a complex post-transcriptional regulation of A1M that should be examined in future studies.

Auto-oxidation of Hb generates superoxide that dismutates into H_2_O_2_. Peroxides facilitate two-electron oxidation of Hb catalyzing FerrylHb formation, while the reaction of OxyHb with peroxides yields FerrylHb radicals (Hb•+(Fe^4+^ = O^2−^)), where the unpaired electron associates with the porphyrin ring or the globin [20,20,67,68,69,70]. This results in strongly reactive high-valence iron compounds that can decay in several ways [69,71,72]. FerrylHb has a transient nature and auto-reduces easily back to metHb through its pseudoperoxidative cycle that damages the globin chains. FerrylHb forms free radicals in the alpha (αTyr-24, αHis-20, αTyr-42) and beta (βTyr-36, βTyr-130) chains of globins [73,74,75]. Besides, βCys-93 irreversibly oxidizes in the presence of oxidants, leading to the destabilization of Hb and the loss of heme [76,77]. Termination reactions of globin-centered radicals catalyze globin–globin crosslinks resulting in Hb dimer, tetramer and multimer formation. In addition to this, H_2_O_2_ is reported to directly react with FerrylHb that is associated with heme degradation [78]. Being a potent oxididant, FerrylHb steals an electron from H_2_O_2_ and reduces back to MetHb, with the concomitant oxidation of H_2_O_2_ to superoxide. This reaction continuously produces superoxides that oxidize the tetrapyrole rings leading heme degradation with iron release [78]. Free iron, if it remains unsequestred, reacts with H_2_O_2_ and aggravates oxidative damage via the Fenton reaction. Overall, FerrylHb generates a highly oxidizing milieu rich in ROS that is likely to amplify FerrylHb-mediated oxidative damage and might induce a more robust A1M expression. This will be further examined in our future study.

Given that Hb oxidation is implicated in the pathophysiology of several diseases with hemolysis, from a therapeutic perspective, it is critical to support endogenous Hb detoxification systems. Here, we showed that A1M significantly inhibited Hb oxidation, revealing a new function of A1M in hemolytic diseases. This is supported by a recent study showing that exogenous A1M is neuroprotective in a preterm rabbit pup model of intraventricular hemorrhage [79]. In addition, A1M or rA1M may have therapeutic benefits in reducing toxicity, due to Hb oxidation during blood transfusion or the application of hemoglobin-based oxygen carriers [80].

Lipid oxidation initiated by ROS plays a significant role in atherosclerosis [46,81] and heme, as a hydrophobic iron complex, behaves as a catalyst in this process [27,28,29,82]. The origin of free heme in the vessel wall is Hb, especially its oxidized forms. Inflammation goes parallel with lipid oxidation in atherosclerosis inflammation, serving as a significant source of ROS to the central pathophysiology of plaque development [83]. More importantly, Hb itself also generates free radicals. Since we have a model to test the cytotoxic effect of LDL lipid peroxidation towards vascular cells, we tested whether A1M could inhibit heme-catalyzed lipid peroxidation initiated by H_2_O_2_ and, in this way, save human aortic endothelial cells. Here, we showed that A1M inhibits heme-driven oxidative modification of LDLs and plaque lipids derived from human atherosclerotic plaques and mitigates oxidized LDL-driven cell death. Thus, it is reasonable to assume that decreasing heme-driven lipid oxidation might have therapeutic significance in atherosclerosis. Previously, it was shown that A1M inhibited myeloperoxidase-induced oxidation of LDL-particles [84]. Myeloperoxidase is another heme protein believed to participate in the development of atherosclerosis, suggesting a general antioxidation role of A1M in the control of atherosclerosis. Importantly, previous studies on protective mechanisms against lipid peroxidation catalyzed by heme and Hb have shown an involvement of the heme-scavenger protein Hpx and Hb-scavenger Hp [82,85,86]. Both Hpx and Hp represent an endogenous protective stratagem against hemolysis-driven endothelial damage [31,87,88]. Given that A1M is a potent heme-scavenger protein with reductase function and antioxidant properties, it represents a potential therapeutic approach to mitigate hemolysis-driven endothelial damage. Albumin itself has heme-binding capacity with low and high affinity binding sites helping the plasma clearance of free heme. However, equimolar concentration to heme did not inhibited the heme-mediated EC sensitization to ROS [89]. In cell cultures, specific heme-binding proteins, such as Hpx, were able to prevent cell sensitization towards heme [90]. Finally, yet another potential anti-atherogenic mechanism of A1M may be the stabilization of RBCs, preventing hemolysis induced by free heme, iron and mechanical as well as osmotic stress [91]. Altogether, this strongly suggests that the protective effects observed by A1M are specific; however, further studies are needed to more precisely clarify the mechanism(s) involved.

In summary, we demonstrated a robust cellular A1M immunoreactivity in the atherosclerotic region of human carotid arteries, especially after plaque hemorrhage, establishing a strong linkage with the progression of atheromas, plaque hemorrhage and the presence of A1M. Cell culture experiments revealed that A1M can be produced locally in atherosclerotic plaques by the resident cells. We showed that FerrylHb is the most prominent inductor of A1M. We also showed, for the first time, that A1M significantly inhibited the oxidative modification of Hb and plaque lipids that may have therapeutic benefit to attenuate oxidized Hb- and lipid-driven toxicity in hemolytic diseases.

## 4. Materials and Methods

### 4.1. Cell Culture

Human aortic smooth muscle cells (HAoSMCs) were purchased from Cell Applications (San Diego, CA, USA, Cat.: 354-05a) and Lonza (Allendale, NJ, USA, Cat.: CC-2571). HAoSMCs were grown in Dulbecco’s Modified Eagle Medium (DMEM) containing 1000 mg/L glucose supplemented with 10% fetal bovine serum (Life Technologies, Vienna, Austria), 100 U/mL penicillin, 100 μg/mL streptomycin and neomycin, referred to as growth medium (GM). HAoSMCs were grown to confluence and used from passages 5 to 7. Human aortic endothelial cells (HAoECs) were obtained from Lonza (Allendale, NJ, USA). HAoECs were grown in EBM endothelial growth medium (Lonza, Allendale, NJ, USA) supplemented with 10% FBS, 100 U/mL penicillin, 100 µg/mL streptomycin and amphotericin B. They were grown to 90% confluence and used from passages 4 to 6. Peripheral blood mononuclear cells (PBMCs) were isolated from healthy volunteers and maintained in Dulbecco’s Modified Eagle Medium (DMEM) containing 1000 mg/L glucose supplemented with 10% fetal bovine serum (Life Technologies, Vienna, Austria), 100 U/mL penicillin, 100 μg/mL streptomycin and neomycin, referred to as growth medium (GM). To examine A1M production, cells were exposed to heme (10 µM), OxyHb (Fe^2+^; 10 µM heme group) and FerrylHb (Fe^4+^; 10 µM heme group) in the corresponding medium with 5% fetal bovine serum, 100 U/mL penicillin, 100 μg/mL streptomycin and neomycin, for different lengths of time, as indicated in the figure legends.

### 4.2. Hb Preparations

Hb of different redox states, that is, OxyHb (Fe^2+^) and FerrylHb (Fe^4+^), was prepared as described previously [25]. Briefly, Hb was purified from fresh blood drawn from healthy volunteers with ion-exchange chromatography on a DEAE Sepharose CL-6B column. To generate FerrylHb, purified Hb was incubated for 1 h at 37 °C with a 10:1 ratio of H_2_O_2_ to heme followed by dialysis against saline (3 times for 3 h at 4 °C) and concentration using Amicon Ultra centrifugal filter tubes (10,000 MWCO, Millipore Corp., Billerica, MA, USA). Aliquots were snap-frozen in liquid nitrogen and stored at −80 °C. The purity of each Hb preparation was measured by SDS-PAGE followed by staining with the ProteoSilver Plus Silver Staining Kit. The purity of Hb preparations was above 99.9%. Hb concentrations were calculated as described by Winterbourn [92]. To examine the effect of rA1M on Hb oxidation, Hb (10 µM) was exposed to 100 µM and 200 µM concentrations of H_2_O_2_ for 3 h at 37 °C in the presence or absence of rA1M (20 µM). The oxidation process of Hb was followed by measuring the oxidation states of the heme-iron by analyzing the absorbance spectra (500–700 nm) of Hbs with a spectrophotometer (Beckman Coulter, Brea, CA, USA). Hb ratios were calculated as described previously by Winterbourn [92].

### 4.3. Analysis of Hb Oxidation by Immunoblot

Hb oxidation was analyzed by immunoblotting, using HRP-conjugated goat anti-human Hb polyclonal antibody (Abcam, Cambridge, UK). After FerrylHb preparation, we analyzed FerrylHb formation by silver staining and Hb-specific immunoblot. In contrast to Hb and MetHb, Ferryl Hb was polymerized as detected by both silver staining and immunoblot, according to previous studies [25,93]. Unfortunately, a limitation of this method is that it does not provide data on the degree of FerrylHb formed during Hb oxidation. Based on these studies, FHb equations are available when the starting is MetHb to quantify exact FHb content [94]. However, in this study we generated FerrylHb by reacting Hb with H_2_O_2_, that does not allow to use this equation. Therefore, we followed FerrylHb formation according to earlier studies by Winterbourn [92] and immunobloting [25,93].

### 4.4. Tissue Samples

Carotid arteries from patients who underwent carotid endarterectomy were obtained from the Department of Surgery at the University of Debrecen. The study was conducted in accordance with the Declaration of Helsinki. The sample collection was approved by the Scientific and Research Ethics Committee of the Scientific Council of Health of the Hungarian Government under the registration number of DE OEC RKEB/IKEB 3712-2012. Written informed consent was received from the participants. Specimens were examined by a trained pathologist and classified according to AHA guidelines. Type I (healthy), IV (atheromatous) and VI (complicated with fresh and organized hemorrhage) lesions were selected for further investigation.

### 4.5. Immunohistochemistry

Carotid artery specimens were fixated with formaldehyde (4%) in phosphate-buffered saline solution pH 7.4 for 1–3 days—based on the size of the sample. Vascular segments were then embedded in paraffin wax, then 3–5 µm thick slides were deparaffinated using xylene and ethanol. After inhibition of endogenous peroxidase (0.5% H_2_O_2_ for 20 min), activity slides were subjected to antigen retrieval in antigen retrieval buffer solution (pH 9.0, RE7119, Leica, Wetzlar, Germany). Samples were incubated with Dako EnVision FLEX Peroxidase-Blocking Reagent (Dako, Glostrup, Denmark) for 5 min in a wet chamber, rinsed with EnVision FLEX Wash Buffer, Tris-buffered saline solution containing Tween 20, pH 7.6 (±0.1). Next, slides were incubated with anti-alpha 1 microglobulin antibody (EPR5880) (Abcam, Cambridge, UK) or CD68 (Agilent Technologies, Santa Clara, 95051 CA, USA) or CD34 (Leica Biosystems, Buffalo Grove, 60089 IL, USA) or smooth muscle α-actin (Agilent Technologies, Santa Clara, 95051 CA, USA) using the ultraView Universal DAB Detection Kit based on protocol No. 5269806001. The intensity and distribution of α_1_-microglobulin-, CD68-, CD34- and smooth muscle α-actin-specific immunostaining was assessed by light microscopy (Leica DM2500 microscope, DFC 420 camera and Leica Application Suite V3 software, Leica). Counterstaining was performed with Gill Hematoxylin solution (105175 Merck Millipore, Billerica, MA, USA).

### 4.6. RNA Isolation and Quantitative Reverse Transcription-Polymerase Chain Reaction

Cells were grown on 24-well plates and total RNA was isolated with TriReagent (Zymo Research, Irvine, CA, USA) and reverse-transcribed using the High-Capacity cDNA Reverse Transcription Kit (Applied Biosystems Inc., Foster City, CA, USA). The gene encoding A1M is designated as α_1_-microglobulin-bikunin precursor gene (AMBP) encoding A1M and bikunin proteins. AMBP and HO-1 mRNA expressions were determined by predesigned TaqMan Gene Expression Assays (Thermo Fisher Scientific, Waltham, MA, USA) and were normalized to GAPDH. Assay IDs for predesigned TaqMan Gene Expression Assays are as follows: AMBP: Hs00155697_m1; HO-1: Hs01110250; GAPDH: Hs02758991_g1. Reverse transcriptions and qPCRs were performed using the C1000 Thermal Cycler with CFX 96 Real-Time PCR System (Bio-Rad, Hercules, CA, USA). Relative mRNA expressions were calculated with the ΔΔCt method using GAPDH as an internal control.

### 4.7. Immunoblot

To analyze A1M secretion into the cell culture medium, equal volumes of cell culture supernatants were electrophoresed on 12% Tris-glycine SDS-PAGE gels, then proteins were transferred to 0.22 µm nitrocellulose membrane (GE Healthcare, Chicago, IL, USA) and blocked with 5% ***w/v*** milk for 60 min; they were then probed for A1M antibody (Abcam, Cambridge, UK; dilution 1:2500) at 4 °C overnight, followed by incubation with horse-radish peroxidase (HRP)-conjugated goat anti-rabbit antibody (Cell Signaling Technologies, Danvers, MA, USA) at room temperature for 1 h. The antigen–antibody complex was detected by a WesternBright ECL HRP substrate (Advansta, Menlo Park, CA, USA). To ascertain equivalent protein loading in the samples, membranes were stripped and reprobed again with a mouse anti-bovine serum albumin antibody (Abcam, Cambridge, UK). To analyze HO-1 expression, cells were lysed with a radioimmunoprecipitation assay buffer (50 mM Tris pH 7.5, 150 mM NaCl, 1% Igepal CA-630, 1% Sodium-deoxycholate, 0.1% SDS, 1 × Complete Mini Protease Inhibitor Cocktail) and analyzed by immunblot, as described above, using HO-1 antibody (Proteintech Group, Manchester M3 3WF, UK; dilution 1:8000), then reprobed with a mouse GAPDH antibody (Proteintech Group, Manchester, UK; dilution 1:10,000).

### 4.8. A1M

Recombinant human A1M (A1M) with an N-terminal His-tag [95] was expressed, purified and refolded from Escherichia coli cultures, as described by Åkerström et al. [96].

### 4.9. Preparation and Oxidation of Low-Density Lipoproteins

Preparation and oxidation of low-density lipoproteins (LDL) was performed as described elsewhere [97]. Briefly, LDL was isolated from the plasma of EDTA-anticoagulated venous blood of healthy volunteers by gradient ultracentrifugation (Beckman Coulter, Inc., Brea, CA, USA). Plasma density was adjusted to 1.3 g/mL with KBr and a two-layer gradient was prepared in a Quick-Seal ultracentrifuge tube by layering saline on 10 mL of plasma. Ultracentrifugation was performed at 302,000 × *g* at 4 °C for 2 h (VTi 50.2 rotor). LDL samples were stored at −70 °C and protein concentration was determined with the Pierce BCA protein assay Kit (Thermo Scientific, Waltham, MA, USA). LDL oxidation was carried out by the addition of heme (5 µM) and H_2_O_2_ (75 µM).

### 4.10. Plaque Lipid Preparation and Oxidation

Carotid artery vessels and complete lesions were removed and immediately washed with physiological saline, dried, weighed, frozen in liquid nitrogen and stored at −70 °C. Lesions with thickened tunica intima and large lipid core without the sign of disruption were used to extract lipids from by chloroform:methanol (2:1 ***v/v***) according to the method of Bligh and Dyer [98]. Plaque lipids (0.5 mg/mL) were incubated with heme (5 µM) in the presence or absence of A1M (10 µM) for 3 days at 37 °C. Lipid peroxidation was assessed by measuring TBARs.

### 4.11. Measurement of Lipid Peroxidation Products in LDL and Plaque Lipids

Measurement of lipid peroxidation products in LDL was performed as described elsewhere [97]. To measure conjugated diene content, samples were diluted to 200 μg protein/mL, followed by optical density measurement at 234 nm. Total LOOH content of LDL was measured as described earlier [99]. To measure thiobarbituric-acid reactive substances (TBARs), 50 μL of a 200 μg protein/mL LDL sample was mixed with 100 μL of thiobarbituric acid reagent (0.375 g 2-thiobarbituric acid, 2.08 mL HCl, 15 mL 10% trichloroacetic acid to a final volume of 100 mL), followed by heating (90 °C for 20 min). Samples were then cooled and extracted with 200 μL of n-butanol. The upper phase was measured spectrophotometrically at 532 nm. Results were calculated using a molar extinction coefficient of 1.56 × 105 M^−1^ cm^−1^ and are expressed as nmol TBARs/mg protein.

### 4.12. Effect of Recombinant A1M on Heme-Induced LDL Oxidation

Native LDL (200 µg/mL) was incubated alone or in the presence of heme (5 µM), heme (5 µM) and H_2_O_2_ (75 µM), or heme (5 µM), H_2_O_2_ (75 µM) and A1M (10 µM) in Hanks’s balanced salt solution with Ca^2+^- ad Mg^2+^ (HBSS+) for 60 min at 37 °C. Conjugated diene, total LOOH and TBARs were measured as described above.

### 4.13. EC Cytotoxicity Assay

Confluent HAoECs grown in 96-well tissue-culture plates were washed twice with HBSS+ and exposed to native LDL (200 µg/mL), native LDL (200 µg/mL) and heme (5 µM), native LDL (200 µg/mL) and H_2_O_2_ (75 µM), or native LDL (200 µg/mL), heme (5 µM) and H_2_O_2_ (75 µM) in the presence or absence of A1M (10 µM) in HBSS+. Cells were then incubated at 37° for 6 h in a CO_2_ incubator. Cell viability was assessed by MTT assay.

## Data Availability

The datasets generated during and/or analyzed during the current study are available from the corresponding author on reasonable request.

## Supplementary Materials

The following are available online at https://www.mdpi.com/article/10.3390/ijms22136668/s1.
