# Supplementary material for: Ferryl Hemoglobin and Heme Induce A1-Microglobulin in Hemorrhaged Atherosclerotic Lesions with Inhibitory Function against Hemoglobin and Lipid Oxidation
Source: Int J Mol Sci. 2021 Jun 22;22(13):6668. doi: 10.3390/ijms22136668 (PMC8268598; doi:10.3390/ijms22136668)
Supplement: Supplementary file 1 [file ijms-22-06668-s001.zip › ijms-1256975-supplementary.pdf]

Supplementary information

Ferryl Hemoglobin and Heme Induce A_1-Microglobulin in Hemorrhaged Atherosclerotic Lesions with Inhibitory Function Against Hemoglobin and Lipid Oxidation

Dávid Pethő [1,2,†], Tamás Gáll [1,3,†], Zoltán Hendrik [4], Annamária Nagy [1,2], Lívia Beke [5], Attila Péter Gergely [4], Gábor Méhes [5], Csaba Tóth [6], Magnus Gram [7], Bo Åkerström [8], György Balla [3,9] and József Balla [1,*]

[1] Division of Nephrology, Department of Internal Medicine, Faculty of Medicine, University of Debrecen, 4032 Debrecen, Hungary; petho.david@med.unideb.hu (D.P.); gall.tamas@med.unideb.hu (T.G.); nagy.annamari90@gmail.com (A.N.)

[2] Kálmán Laki Doctoral School, Faculty of Medicine, University of Debrecen, 4032 Debrecen, Hungary

[3] ELKH-UD Vascular Biology and Myocardial Pathophysiology Research Group, Hungarian Academy of Sciences, University of Debrecen, 4032 Debrecen, Hungary; balla@med.unideb.hu

[4] Department of Forensic Medicine, Faculty of Medicine, University of Debrecen, 4032 Debrecen, Hungary; dr.hendrik.zoltan@gmail.com (Z.H.); gergely.peter@med.unideb.hu (A.P.G.)

[5] Department of Pathology, Faculty of Medicine, University of Debrecen, 4032 Debrecen, Hungary; beke.livia@med.unideb.hu (L.B.); gabor.mehes@med.unideb.hu (G.M.)

[6] Division of Vascular Surgery, Department of Surgery, Faculty of Medicine, University of Debrecen, 4032 Debrecen, Hungary; toth.csaba@med.unideb.hu

[7] Department of Clinical Sciences Lund, Pediatrics, Lund University, 22184 Lund, Sweden; magnus.gram@med.lu.se

[8] Department of Clinical Sciences Lund, Infection Medicine, Lund University, 22184 Lund, Sweden; bo.akerstrom@med.lu.se

[9] Department of Pediatrics, Faculty of Medicine, University of Debrecen, 4032 Debrecen, Hungary

* Correspondence: József Balla balla@belklinika.com; Tel.: +36-52-255-500 (ext. 55004)

† Authors contributed equally.

Supplementary figure

A

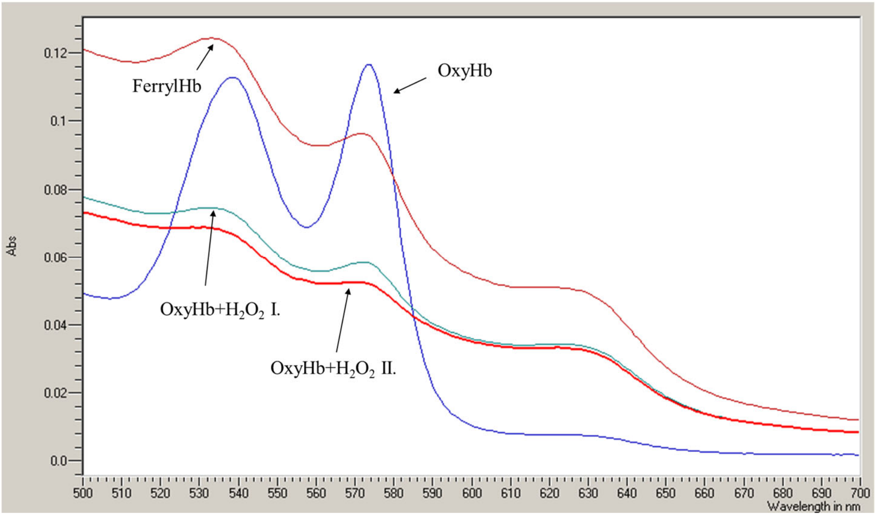

B

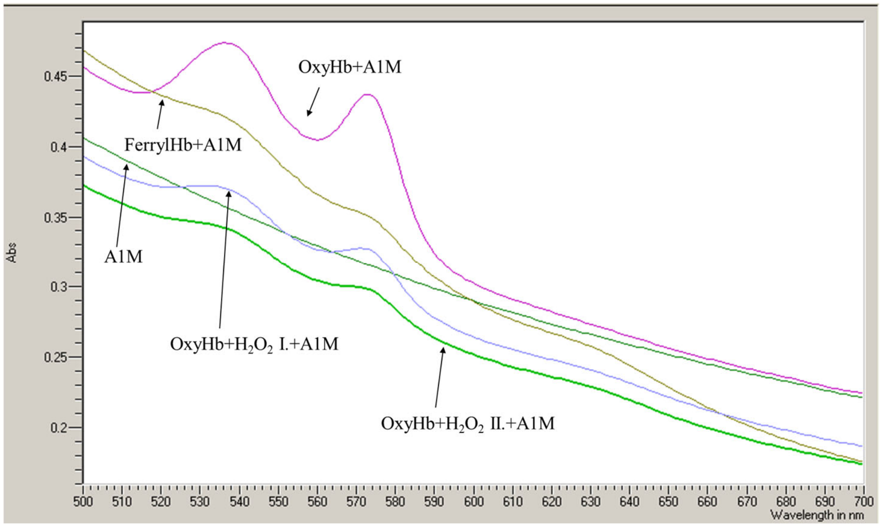

Supplementary Figure S1. Absorption characteristics for the different Hb-oxidation states. Purified OxyHb (10 µM) was incubated for 3 h at 37°C with a 100 (OxyHb+H_2O_2 I.) or 200 µM (OxyHb+H_2O_2 II.) of H_2O_2 in the presence or absence of rA1M (20 µM). Absorbance spectra (500-700 nm) of Hbs were taken with a spectrophotometer (Beckman-Coulter, Brea, CA, US) and Hb ratios were calculated as described previously by Winterbourn. **(A)** Reactions of OxyHb with H_2O_2. **(B)** Reactions of OxyHb with H_2O_2 and rA1M.
